# Supplementary material for: Frequent pauses in Escherichia coli flagella elongation revealed by single cell real-time fluorescence imaging
Source: Nat Commun. 2018 May 14;9:1885. doi: 10.1038/s41467-018-04288-4 (PMC5951861; doi:10.1038/s41467-018-04288-4)
Supplement: Supplementary file 1 — Supplementary Information [file 41467_2018_4288_MOESM1_ESM.pdf]

Supplementary Information (SI)

Frequent pauses in *Escherichia coli* flagella elongation revealed by single cell real-time fluorescence imaging

Zhao, *et al*.

Supplementary Note 1: Examine the effects of the tetracysteine tag insertion. Although the tetracysteine tag (TC tag) is small in size, careful examination was performed to investigate the potential influence of the TC tag on cell growth and flagellar function. Saturated bacterial cells of the TC tag inserted strain (RP437-TC-FliC) and wild type strain were transferred into fresh LB medium and cell aliquots of 200 µl were measured for optical density (OD_{600}) at 1-hour time intervals. As presented in Supplementary Fig. 1a, the identical growth curves for the two strains indicate that TC tag insertion does not affect cell viability. In addition, the bacterial mobility of the two strains was also checked on soft Bacto agar plates containing 0.45% (w/v) agar mixed with LB medium. 2.5 µl overnight cultures of each strain were inoculated on the swim agar plates. Plates were incubated overnight face up at 30°C and the motility halo diameters were measured. The collective swimming motility of the TC strain was similar to that of the wild-type strain (Supplementary Fig. 1b), suggesting that the introduction of the tetracysteine tag has little effect on bacterial mobility.

Supplementary Note 2: Examine the effects of FlAsH labeling. In our experiments, FlAsH was kept in the culturing medium to ensure timely labeling of the flagella for long-term observation under a microscope. Considering the potential toxic effect of FlAsH, we compared the optical density and flagellar lengths between *E. coli* cells cultured with and without FlAsH. Saturated cell cultures were inoculated into fresh

M9 medium (with or without FlAsH) and cell aliquots of 200 μl were measured for optical density (OD_{600}) at 1-hour time intervals. Supplementary Fig. 2a shows that cells had identical growth curves, regardless of whether FlAsH was added, suggesting that FlAsH labeling does not affect cell growth. For the experiment of flagellar length measurement, the culturing and labeling procedures were performed following the methods described in the main text. Saturated cell cultures were inoculated in fresh M9 medium (with or without FlAsH) to incubate for 4, 5, 6 and 7 hours. The cells grown without FlAsH were stained only before flagellar length measurements. As shown in Supplementary Fig. 2b, the distributions of flagellar lengths in the two culture groups at different incubation times were similar, even when they were grown in the presence of FlAsH as long as 7 hours, demonstrating that flagellar growth is not affected by FlAsH labeling.

Supplementary Note 3: Flagellar growth rate measurements by real-time imaging based on population data. To confirm the inverse correlation between flagellar elongation rate and length, we performed time-lapse fluorescence imaging to record flagellar growth of *E. coli* flagella at different lengths. Briefly, saturated cultures were inoculated at 1:100 dilutions into M9 medium to an OD_{600} of approximately 0.3-0.4 and then transferred under the microscope to monitor flagellar growth (Methods). Images were taken every 10 min. Repeat centrifugation, which causes flagellar breakage, was avoided. Supplementary Fig. 3a shows representative

time-lapse images of the stained flagellum of the same cell at time T and $T + \Delta T$ with the lengths L and $L + \Delta L$. All flagella chosen for analysis were not tangled with other flagella and the bases of flagella are clearly imaged. Supplementary Fig. 3b shows 338 data points from 102 cells with flagellar lengths ranging from 216 nm to 6547 nm. The instantaneous flagellar growth rate vs. flagellar length is plotted in blue dots. A box plot was constructed to show the dispersion of growth rates at 1 μm binned flagellar lengths (Supplementary Fig. 3c). As a whole, *E. coli* flagella displayed different growing speeds reaching from 0 nm/min to 58.39 nm/min, and even filaments of similar lengths grew at varying rates. Furthermore, high speed was also observed in longer flagellar lengths, indicating that flagella do not grow at a constant rate as formerly reported, and that the flagellar growth rate is not simply dependent on flagellar length. Additionally, the average growth rates showed a decrease from ~ 16 nm/min to approaching 0nm/min as flagella grew longer. Together, these results show that in *E. coli*, the elongation rate of flagella decays with large fluctuation during flagella formation.

Supplementary Note 4: Non-tethered flagella growth rate measurement. To further prove that the observed pause in flagellar growth is not an artifact caused by attaching flagella to poly-l-lysine on the surface, we conducted a similar experiment with cells that are immobilized on an agarose pad when the flagella are free to move in space. Briefly, saturated cultures were inoculated at 1:100 dilutions into M9

medium to an OD_{600} of approximately 0.3-0.4 and then transferred onto a 0.5% (w/v) agarose pad under the microscope to monitor flagellar growth. Images were taken every 10 min. Supplementary Fig. 4a shows two representative examples of single flagellar growth. Because of the strong background of gel pad and the movement of flagella, we can only analyze very limited cells in which accurate flagellar length measurement is possible. The growth measurement of 9 individual flagellum was displayed in Supplementary Fig. 4 b-d. We see that although we have a limited amount of data, we are still able to observe pausing in flagellar growth, confirming our earlier conclusion that it is an intrinsic feature of *E. coli* flagellar growth.

Supplementary Note 5: Examination of cell physiology. As noted in Methods, poly-l-lysine was used to immobilize cells in our time-lapse fluorescence imaging; and cells were centrifuged and washed in the double- and triple-color labeling. To check the effects of poly-l-lysine and washing steps on the physiology of bacteria, we have performed additional experiments to measure the bacterial growth in both condition.

For poly-l-lysine issue, we conducted a similar experiment with *E. coli* cells that are immobilized on an agarose pad. Briefly, saturated cultures were inoculated at 1:100 dilutions into M9 medium to an OD_{600} of approximately 0.3-0.4 and then transferred to a 0.5% (w/v) agarose pad or a poly-l-lysine coated tunnel. Cells then were observed under the bright-field microscope to monitor bacterial growth for several hours.

Images were taken every 10 min. In supplementary Fig. 5a, representative images of cell growth on poly-l-lysine coated tunnel (top) and gel pad (bottom) were displayed. For each group, the doubling time of 100 cells were measured and showed in supplementary Fig. 5b, both of which are about 50 min on average. This results revealing that 1-min surface coating by 0.01% poly-l-lysine did not affect cell growth.

Next, we compared the bacterial growth in double-color labeling procedures with and without washing step. We cultured *E. coli* cells in M9 medium for 4 hours and diluted the culture to an appropriate concentration and labeled with 2.5 μM ReAsH. For unwashed group, cells were imaged directly by bright-field microscopy to record bacterial growth for several hours. For washed group, cells were washed by centrifugation for 5 min at 1400 x *g* then observed under the microscope. For each group, 100 cells were measured and the doubling time of bacteria are shown in Supplementary Fig. 5c. No significant difference was found between the two groups, indicating that washing steps did not affect the physiology of the cells.

Supplementary Note 6: Flagellin content measurement. To rule out the possibility that the observed pause in flagellar growth rate was caused by different culturing medium, we conducted a measurement by single-cell fluorescence imaging and immunoblotting to estimate the amount of flagellin in *E. coli* cells.

Saturated bacterial cultures of the RP437-TC-FliC strain were inoculated into fresh LB and M9 medium and grown to an optical density (OD_{600}) of ~0.3. For single-cell

fluorescence measurements, flagella of bacterial cells were sheared by passing them 5 times in and out of a 20-ml syringe equipped with a 27-gauge needle and washed away by centrifugation. For both groups, we centrifuged cells at 1400 x *g* for 5 min and resuspended the cell pellets in M9 medium containing 10 mM tris(2-carboxyethyl)phosphine (TCEP, Sigma–Aldrich) and 2.5 μM FlAsH. After FlAsH staining and fluorescence imaging, fluorescence intensity in single cells, which rep-resents the intracellular amount of flagellin proteins, were measured with ImageJ. As shown in Supplementary Fig. 7a, the fluorescence intensity of most cells were within the range from 5000 A.U. to 15000 A.U., and pairwise nonparametric t-tests showed no significant differences between the cells grown in LB and cells grown in M9 medium. Immunoblotting of total amounts of flagellin was also performed as a complementary approach following the protocol described in the Methods. The similar amount of flagellin in two cultures was presented in Supplementary Fig. 7b. Together these results show that flagellin expression of *E. coli* cells would not be affected by different growth media.

Supplementary Note 7: Validation of FliC overexpression. In Figure 5b, the increase in the level of overexpressed flagellins seems low, which may because that the total amount of flagellin of bacterial cells was detected, including intracellular flagellins and extracellular filaments. Considering that the assembled filaments outside the cell carry substantial flagellin proteins, the overexpressed flagellins only

account for a very small fraction of the total flagellins and therefore do not result in a large increase in the blotting experiments. To verify this, we removed flagella by shearing before performing western blotting and examined intracellular flagellins. As shown in Supplementary Fig. 8 (relative flagellin levels report Mean ± S.D., n = 3), for intracellular flagellin (two lanes on the right), the increase by overexpression is roughly 2-fold, which is apparently much higher than the total flagellins (two lanes on the left). Despite removing assembled external flagella by shearing, there still remained some truncated external flagella that affected the total amount of flagellin measured using blots.

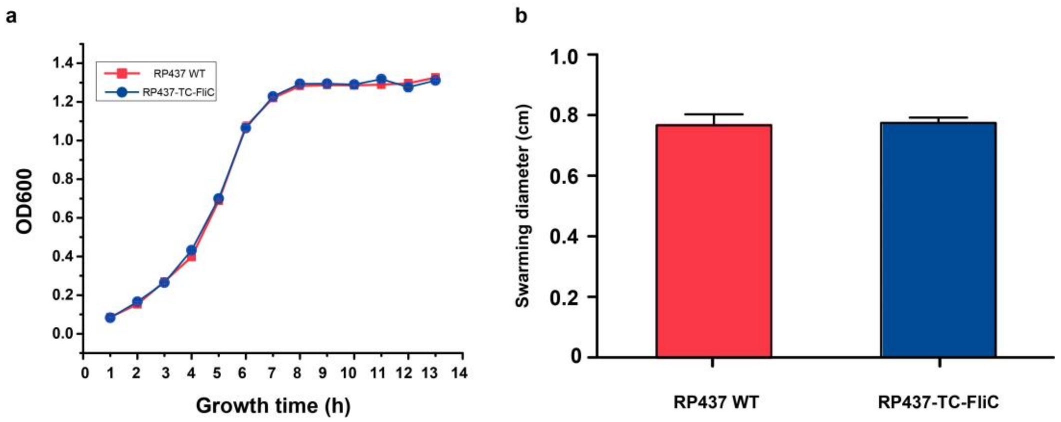

Supplementary Fig. 1 Comparison of the tetracysteine tag inserted strain (RP437-TC-FliC) and wild type strain. a Growth curves in LB medium; **b** Swimming motilities on soft Bacto agar plates containing 0.45% (w/v) agar mixed with LB medium. Mean ± SD: Red, 0.77 ± 10.9 cm; Blue, 0.78 ± 5.8 cm.

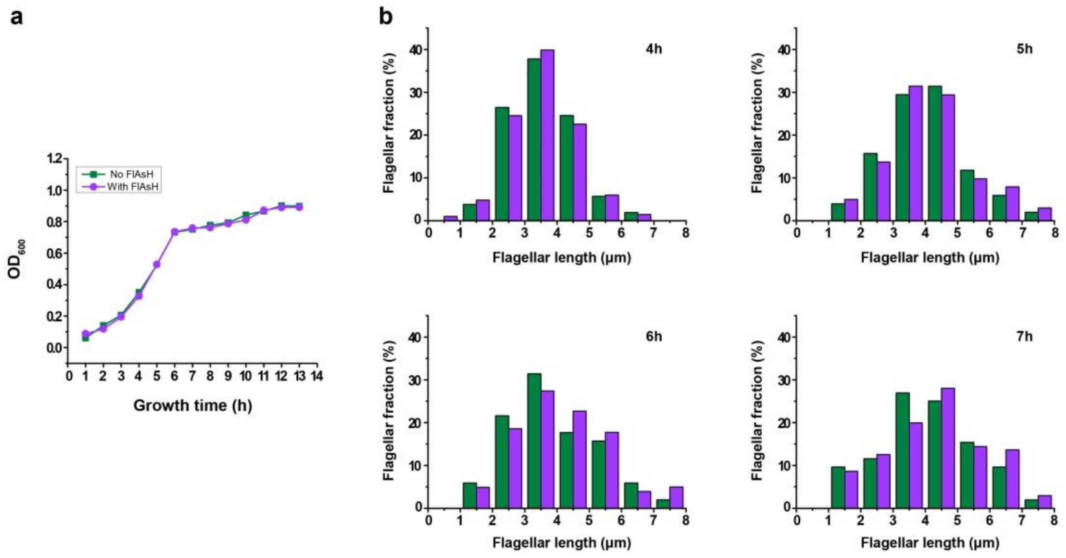

Supplementary Fig. 2 FlAsH labeling does not affect cell viability nor flagellar growth. a Growth curves for the RP437-TC-FliC strain grown in M9 medium with (Purple) or without FlAsH (Green). **b** Distributions of flagellar lengths on cells of the RP437-TC-FliC strain cultured in M9 medium with (Purple) or without FlAsH (Green) at different culture times. The number of flagella that analyzed was 123, 121, 117, and 135 for the strain grown without FlAsH and 127, 109, 134, and 108 for the strain grown with FlAsH.

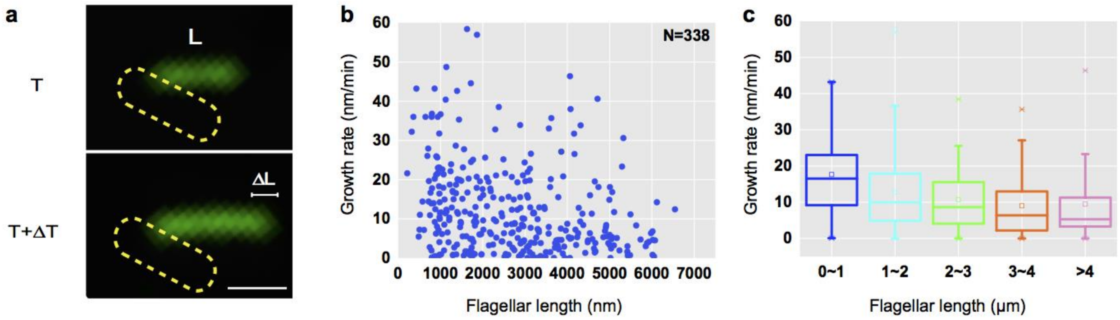

Supplementary Fig. 3 Flagellar growth rate measurements by real-time imaging based on population data. a Representative images of a growing bacterial flagellum at two different time points, $\Delta T = 10$ min. The flagellar length increases by length L in two dimensions. The growth rate of the flagellum can be calculated as $\Delta L / \Delta T$. Scale bar, 1 μm. **b** The growth rates for different flagellar lengths (338 data points from 102 cells) of *E. coli* at 10-min intervals are measured. **c** Box plot of flagellar growth rates from the data in (**b**), binned at 1 μm interval.

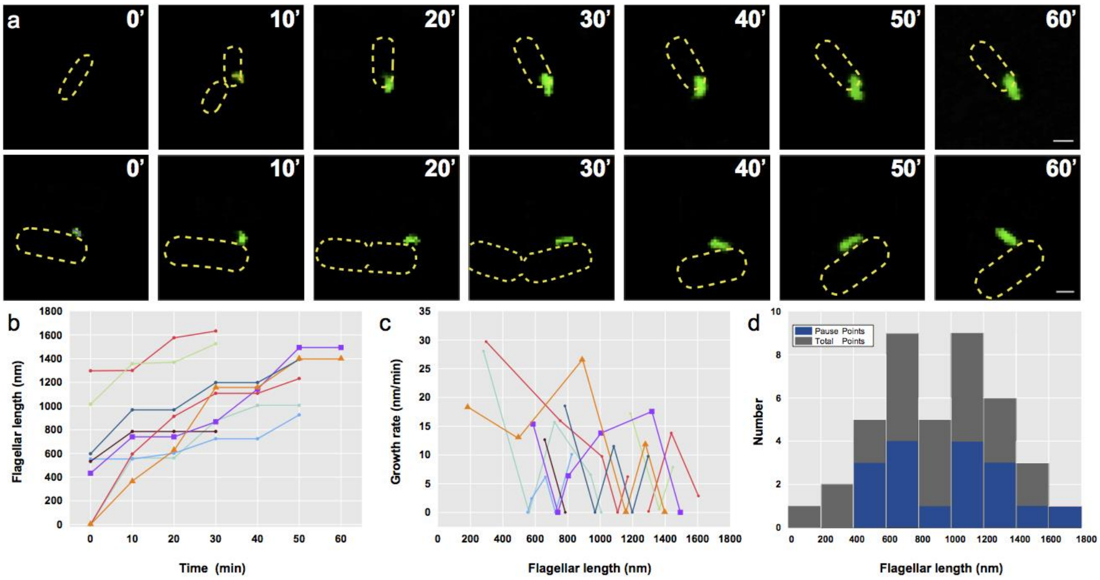

Supplementary Fig. 4 Flagellar growth rate measured from *E.coli* cells immobilized using the agarose pad. a Representative images of two single growing bacterial flagellum of cells immobilized on the agarose pad, taken at time points with 10-min time interval. Scale bar, 1 μm. **b, c** Growth measurements of 9 individual flagella tracked by real-time imaging. Plots of flagellar length versus time (b) and the flagellar growth rates versus flagellar lengths (c) are displayed. The flagella in (a) are emphasized by orange line with triangles (Top) and purple line with squares (Bottom). **d** Distribution of pause events (Blue) occurring in the total collected data points (Dark gray) at different flagellar lengths, binned at 200-nm intervals. All data points are from the measurements in (c).

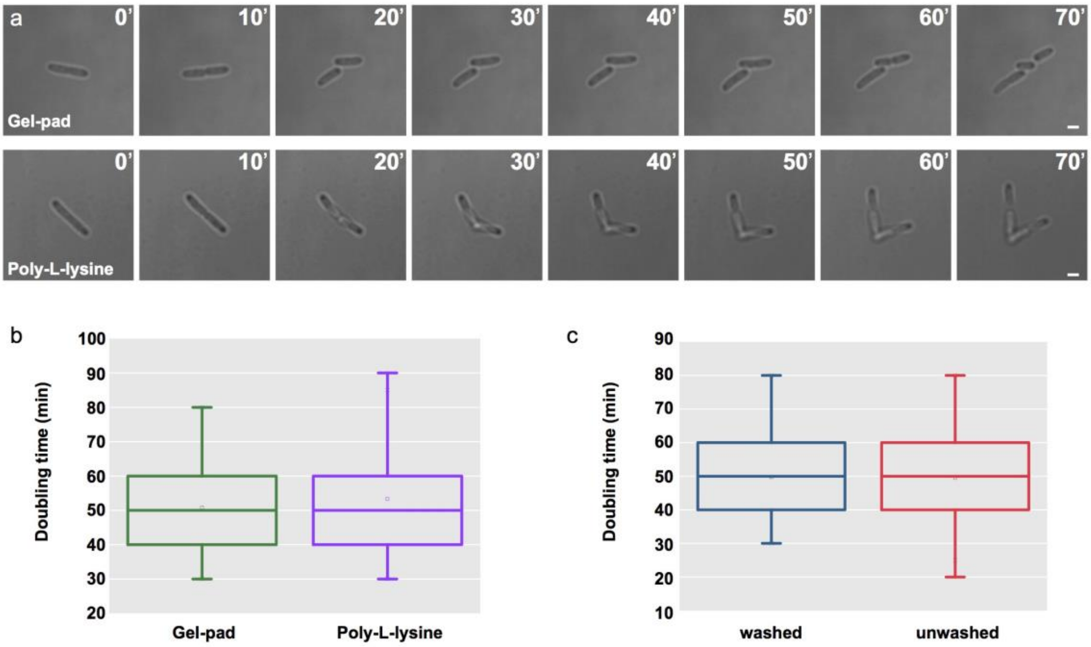

Supplementary Fig. 5 Examination of cell physiology. a Time-lapse bright-field images of bacterial growth recorded on gel pad (Top) and poly-L-lysine (Bottom). Scale bar, 1 μm. **b** Doubling time comparison of cells grown on gel pad (Green) and poly-L-lysine (Purple). 100 cells for each group were analyzed. Mean ± SD: Green, 50.8 ± 10.9 min; Purple, 53.3 ± 12.8 min. Statistical analysis of two-tailed t-test was performed defining difference as insignificant, $P= 0.439$. **c** Doubling time comparison of cells washed by centrifugation (Blue) or without washing (Red). Both washed and unwashed groups were performed on poly-L-lysine coated surface. 100 cells for each group were analyzed. Mean ± SD: Blue, 49.7 ± 11.1 min; Red, 49.5 ± 13.7min. Statistical analysis of two-tailed t-test was performed defining difference as insignificant, $P = 0.908$.

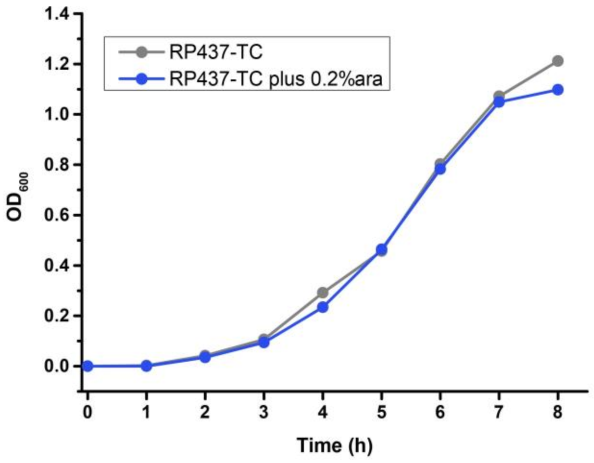

Supplementary Fig. 6 Overexpression of flagellin does not affect cell growth. Growth curves for two cultures (RP437-TC-FliC strain with or without plasmid pBAD18-TC-FliC) possessing different amounts of flagellin induced by the addition of arabinose.

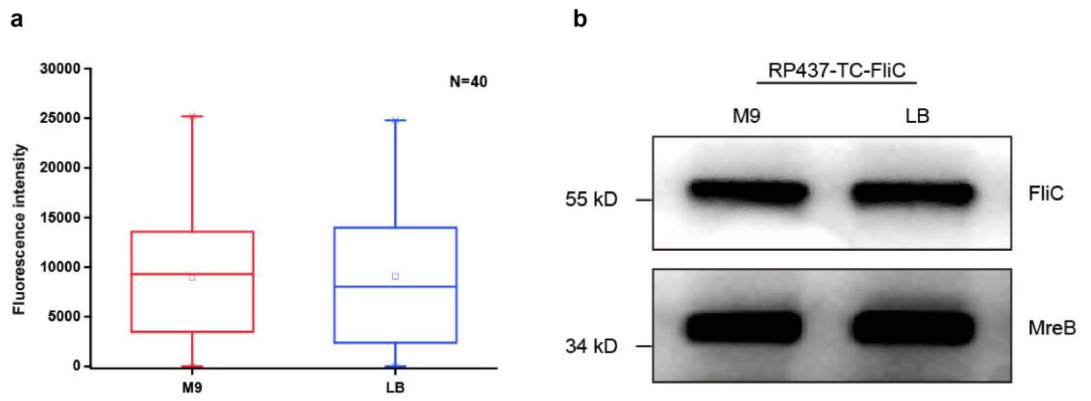

Supplementary Fig. 7 Flagellin measurements. a Single-cell fluorescence (arbitrary unit) measurement with FlAsH labeling and **b** immunoblotting to estimate the cellular flagellin content. No notable difference of intensity was found between the TC strain cells cultured in LB medium and M9 medium. For the single cell assay, 20 cells of each group were measured. Full blots are shown in Supplementary Fig. 9b.

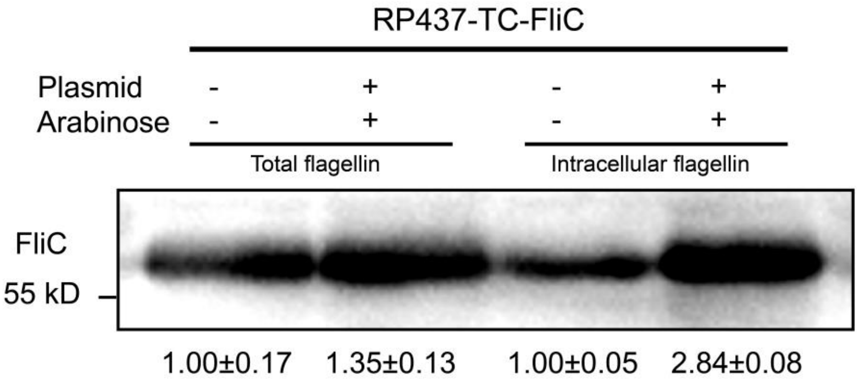

Supplementary Fig. 8 Validation of FliC overexpression. Immunoblotting of total flagellin (Left) and intracellular flagellin (Right) of RP437-TC-FliC strains with or without plasmid expression. The bands were quantitated by ImageJ (relative flagellin levels report Mean ± S.D., n = 3). Full blots are shown in Supplementary Fig. 9c.

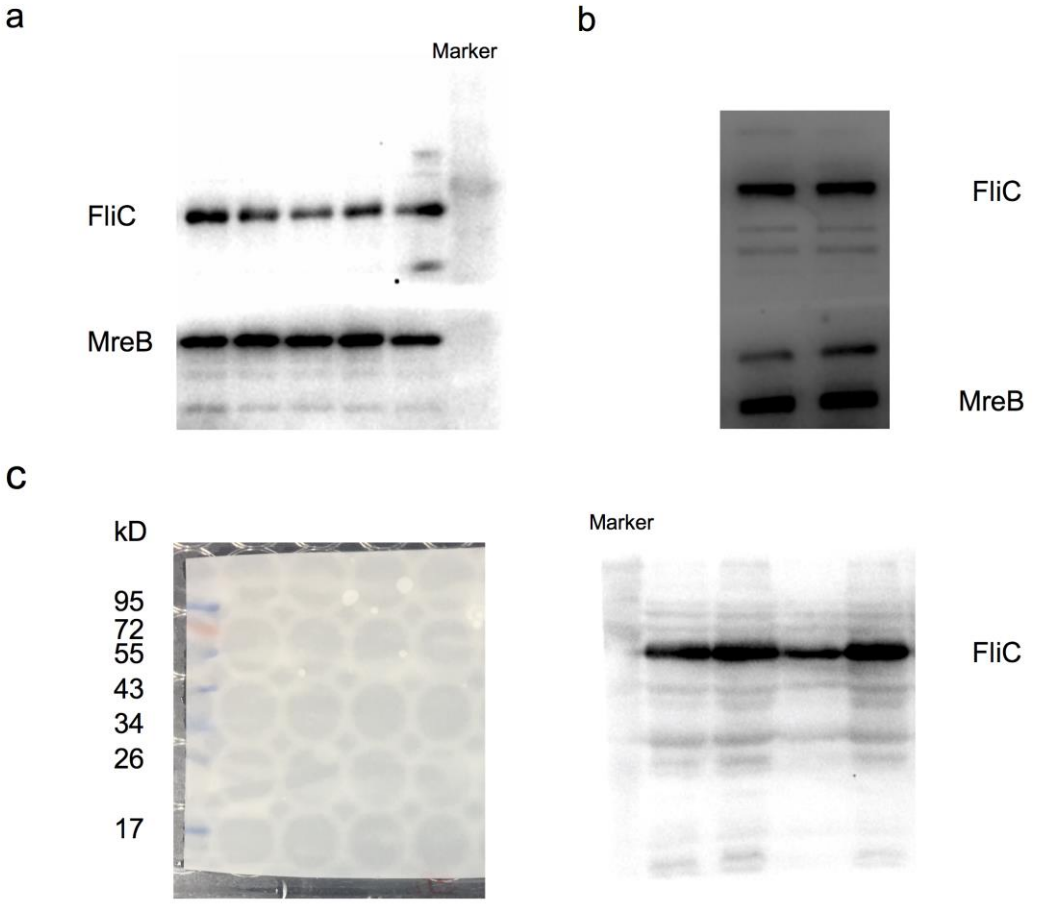

Supplementary Fig. 9 Full blot images of Figure 5b (a), Fig.S7b (b), and Fig. S8 (c).

Supplementary Table 1. List of strains

Strain	Description	Source
RP437 (Wild type)	CGSC 12122	*E. coli* Genetic Stock Center
RP437-TC-FliC	fliC gene chromosomally inserted with TC tag	This study
RP437-pBAD18	Contains plasmid pBAD18-TC-FliC for flagellin expression	This study

Supplementary Table 2. List of primers

Gene	Usage	Forward Primer	Reverse Primer
TC-FliC	TC for pSCS3V31c-Gibson	tttctgaactgttgtcctggctgttgcatggaaccgtctagagcgacgccagacggatttg	cggttccatgcaacagccaggacaacagttcagaaaagaaccgccggatccaccactagtc
	TC-FliC-CmR-Recombination	gatgcaaatactactaaagctacaactatcacttcag<br>gcggtacatttctgaactgttgtcctggctgttgcatggaaccgtc	aaggttggcagttgcggaacctgcagtattatcaatctgaacaggccaccgctcgtctgccgagctc
	TC-FliC	caactatcacttcaggcggtacatttctgaactgttgtcctggctgttgcatggaaccgcctgttcagattgataatactg	gcatatgtatcggtatcattaccctt
pBAD18-TC-FliC	TC-FliC for pBAD18	tagcaggaggaattcaccatggcacaagtcattaataccaacagc	tactttctagagaataggaacttcttaaccctgcagcagagacag
	TC-pBAD18-Backbone	gaagttcctattctctagaaag	catggtgaattcctcctgctagcc
